# Oxidants and Cardiorenal Vascular Remodeling—Insights from Rare Genetic Tubulopathies: Bartter’s and Gitelman’s Syndromes

**DOI:** 10.3390/antiox12040811

**Published:** 2023-03-26

**Authors:** Luca Sgarabotto, Verdiana Ravarotto, Lucia Federica Stefanelli, Martina Cacciapuoti, Paul A. Davis, Federico Nalesso, Lorenzo A. Calò

**Affiliations:** 1 Nephrology, Dialysis and Transplantation Unit, Department of Medicine, University of Padova, 35128 Padova, Italy; 2 Department of Nutrition, University of California, Davis, CA 95616, USA

**Keywords:** oxidants, cardiovascular remodeling, renal remodeling, Bartter’s syndrome, Gitelman’s syndrome

## Abstract

Two human genetic tubulopathies, Bartter’s (BS) and Gitelman’s (GS) syndromes, have normo/hypotension and absent cardiac remodeling despite their apparent angiotensin system (RAS) activation. This seeming contradiction has led to an extensive investigation of BSGS patients, the result of which is that BSGS represents a mirror image of hypertension. BSGS’s unique set of properties has then permitted their use as a human model to probe and characterize RAS system pathways and oxidative stress in cardiovascular and renal remodeling and pathophysiology. This review details the results using GSBS patients that provide a deeper understanding of Ang II signaling and its associated oxidants/oxidative stress in humans. By providing a more complete and complex picture of cardiovascular and renal remodeling pathways and processes, studies of GSBS can inform the identification and selection of new targets and therapies to treat these and other oxidant-related disorders.

## 1. Introduction

Bartter’s (BS) and Gitelman’s (GS) syndromes are two rare human genetic tubulopathies. They are clinically characterized by hypokalemia, normo/hypotension, metabolic alkalosis, hypercalciuria or normocalciuria (in BS) or hypocalciuria (in GS), muscle weakness, polyuria, failure to thrive, polydipsia and other syndrome-specific side effects [1,2]. These syndromes are the result of impaired potassium reabsorption in the kidney, specifically in the thick ascending limb (TAL) and the distal convoluted tubule (DCT), due to mutations of genes encoding the ion channels and cotransporter involved in nephron’s electrolyte trafficking [1,2]. This altered electrolyte trafficking gives rise to renin-angiotensin system (RAS) overactivation. However, strikingly, these patients have normo- or hypotension and do not exhibit the cardiovascular–renal remodeling classically associated with arterial hypertension [3,4]. Furthermore, in addition to the activation of multiple pathways in RAS, like those found in hypertension, they unexpectedly exhibit increased antioxidant defenses, as well. Given these seeming paradoxes, BSGS patients represent an extraordinary opportunity in a human model to uncover and assess potential interventions of RAS- and oxidative stress (OxSt)-related cardiovascular and renal remodeling pathways [3,4].

Nature has provided in BSGS a model showing a blunting and/or reversal of the deleterious effect of increased Ang II-based RAS and ROS signaling (hypertension, cardiac remodeling, etc.). The results from BSGS patients should be useful for identifying targets using BSGS results as a guide to explore potential treatments in patients with hypertension, cardiac remodeling, etc., that mimic the positive effects noted in BSGS. In other words, understanding why BSGS patients do not develop hypertension and its long-term complications and do not present OxSt despite high Ang II and the activation of RAS may give insights into the molecular bases of hypertension and its complications and might provide clues for targets of therapy in hypertension and its long-term complications such as cardiovascular and renal remodeling.

## 2. Gitelman’s and Bartter’s Syndromes

Gitelman’s syndrome was first described in 1966 as a familial disorder characterized by electrolyte disturbances with concomitant hypomagnesemia and hypokalemia [5]. GS’s clinical features are like those induced by the side effect of thiazide’ diuretics therapy, with salt craving, hypotension and muscle cramps. The prevalence of the disease is 1/40,000, making it one of the most common tubulopathies [2]. GS is often quite difficult to diagnose as it requires a high level of clinical suspicion. In fact, a major portion of the population might be affected without knowing [6]. Several mutations have been found in the SCL12A3 gene which encodes for the sodium chloride cotransporter (NCC) in the DCT (Table 1) [7].

The prevalence of BS is 1/1,000,000 and the clinical features to a large extent overlap those of GS, e.g., muscle weakness, anorexia, polydipsia, polyuria, failure to thrive and growth retardation with salt-wasting [1]. A specific feature that distinguishes BS from GS is its very early presentation with growth retardation and failure to thrive [1]. Moreover, during pregnancy, it can produce polyhydramnios and induce premature delivery. Hypercalciuria is another consequence of the excessive sodium wasted, which increases the risk of nephrocalcinosis. There are multiple subtypes of Bartter’s syndrome (Table 1) that are related to the specific gene affected [1] (Table 1).

## 3. ROS, Oxidative Stress and Renin Angiotensin System Activity

Oxidative stress is the loss of intracellular redox homeostasis when the equilibrium between antioxidant and pro-oxidant factors is altered [8]. Reactive oxygen species (ROS) usually refers to free radicals and other non-radical intermediates which quickly react with all the surrounding molecules [9,10]. Superoxide (O_2_^−^•) is among the most prominent ROS species, as O_2_^−^•, given its relative stability, is able to cross membranes, where it not only acts as a signaling molecule but undergoes catalytic decomposition to •OH, an extremely reactive radical [11,12]. Other reactive radical compounds include radical nitric oxide (NO•) and peroxynitrites (ONOO−) [11]. ROS overproduction occurs mainly in mitochondria to produce ROS-driven damage to cellular lipid, proteins and DNA. In addition to direct damage, ROS alter cellular signaling to activate the inflammatory system as well as causing programmed cell death [13]. Another example is that ROS generated by vascular NOX modulates nitric oxide (NO) bioavailability, thereby regulating blood pressure [14].

In normal kidneys, ROS produced through NOX3 controls Na^+^ transport, tubuloglomerular feedback and renal oxygenation [15,16,17]. Furthermore, oxygen radicals increase the loop of Henle’s NaCl absorption, modulating Na^+^/H^+^ exchange [8,9]. Pulmonary NOX2 has been implicated in airway and vascular remodeling [9]. The p22^phox^-dependent NOX2 regulates the proliferation [10] and differentiation [11] of smooth muscle cells through the activation of nuclear factor kappa B (NF-κB) and inducible nitric oxide synthase (iNOS). NOX family members NOX 1–3 require p22^phox^ alongside the recruitment of cytosolic subunits, whereas NOX4 only requires p22^pho^. All share a homologous NOX2-like catalytic subunit responsible for electron transfer and ROS generation [18,19].

Among ROS protective mechanisms, hypoxia inducing factors (HIF) are particularly important. HIF regulate the NOS system, which in turn stimulates HO-1 and Cyclooxygenase 2 (COX2) [20] and the nuclear factor E2 associated factor (Nrf2) system to deal with the ROS overproduction [21]. HO-1 has a very low basal expression but increases rapidly upon oxidative stress. In addition, HO-1 mediates the production of carbon monoxide CO, a known vasodilator, thereby contributing to the regulation of vascular tone, blood pressure and endothelial function [18].

Oxidative stress is a key factor in the pathological progress of atherosclerosis, which underlies cardiovascular diseases such as ischemic heart diseases, stroke and peripheral arterial diseases. Oxidative stress drives endothelial dysfunction by altering endothelial signal transduction and redox-regulated transcription factors. This then increases vascular endothelial permeability and favors leukocyte adhesion [22]. The increase in endothelial permeability allows LDL access to the vessel wall, where they are oxidized to oxLDL [23]; oxLDL are then engulfed by macrophages, becoming foam cells. The presence of foam cells alongside elevated levels of ROS and reduced NO levels promote muscle hyperplasia and dysregulated intima structures such as vascular calcifications. Critically, endothelial damage caused by free radicals results in a vicious circle as damage to the endothelium promotes the further production of free radicals [24,25].

RAS participates in the regulation of a plethora of systems, including blood pressure, fluid and electrolyte balance and systemic vascular resistance. Ang II is a major RAS signaling component whose Janus-faced activity can promote either vasoconstriction, inflammation, fibrosis and cellular growth, or vasodilation, insulin sensitivity, anti-remodeling, and anti-atherogenesis effects [15]. The RAS signaling cascade results from the binding of Ang II with its cell-surface Ang II subtype-1 or subtype-2 receptors (AT1R, AT2R). AT1R receptors are members of the seven transmembrane-domain G protein-coupled receptors superfamily, and their binding promotes the coupling of G proteins (G_q_/G_11_ and/or Gi/G_0_) to the C-terminal domain of the receptor, stimulating many intracellular signaling pathways for PLC, the activation of Ca^2+^ channels, PLA_2_, adenylate cyclase, MAP kinases, the JAK-STAT pathway and NADPH oxidase (NOX), amongst others [15,17]. Given the critical role of Ang II signaling in the regulation of vascular tone, altered Ang II signaling plays a central role in cardiovascular–renal pathophysiology. Persistent activation of the RAS in healthy individuals leads to hypertension and target organ damage. This damage results from both short and long-term effects of Ang II, thus making the short and long-term signaling pathways of Ang II important subjects of investigation to understand, at a molecular level, the mechanisms that cause hypertension and cardiovascular–renal remodeling [15].

**Figure 1:**
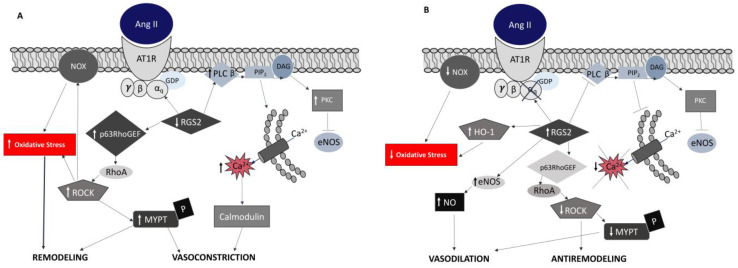
Angiotensin II signaling in chronic kidney disease and hypertension versus angiotensin II signaling in GSBS [3,4,15]. Panel (**A**). Angiotensin II signaling in chronic kidney disease and hypertension promotes through oxidative stress vasoconstriction, vascular remodeling and insulin resistance. Ang II, angiotensin II; AT1R, angiotensin II receptor 1; PLC β, phospholipase C β; PIP2, phosphatidylinositol diphosphate; DAG, diacylglycerol; PKC, protein kinase C; eNOS, endothelial nitric oxidase; NOX, NAD(P)H oxidase, ROCK, Rho Kinase; MYPT, myosin phosphatase protein target. Panel (**B**). Angiotensin II signaling in GSBS is blunted at post-receptor level, thereby promoting vasodilation and insulin sensitivity through RGS-2 activity. RGS, regulators of G-protein signaling; HO-1, heme-oxygenase 1; NO, nitric oxide.

For example, Ang II signaling in chronic kidney disease (CKD) produces alterations in the vasculature as well as in the heart, such as cardiovascular remodeling and hypertension [18] (Figure 1A) [25]. Endothelial dysfunction and atherosclerotic changes are prominent features of CKD even at the very early stages [18]. Endothelial and vascular smooth muscle cells (VSMCs) crosstalk in the context of increased ROS, IL-1 and decreased levels of NO, which characterize CKD and promote VSMCs migration toward intima, causing hyperplasia vascular calcification and inducing arterial stiffness [26,27]. These mediators can also affect the function of epithelial cells of renal tubules and promote epithelial-to-mesenchymal cells transition (EMT), causing kidney fibrosis [14,27].Overall, the RAS, and Ang II as its major effector, are involved in cardiovascular–renal remodeling in CKD by driving oxidative stress and inflammation [17].

A frequent CKD comorbidity is atrial fibrillation (AF), a tachyarrhythmia with irregular and rapid heart rate, which occurs alongside heart failure with preserved ejection fraction and poorly controlled arterial hypertension [28]. There are multiple mechanisms through which ROS can potentially stimulate AF, by causing increased ion leak throughout mycardiocytes [26], as well as by stimulating collagen deposition and fibrosis that interferes with the propagation of the electric impulse [29]. Ang II increases Rho kinase (ROCK) activity, leading to elevated myosin phosphatase target protein subunit-1 (MYPT-1) phosphorylation, and to the increased expression of Connexin 40, an integral membrane protein of heart gap cells junction, which enhances atrial vulnerability to electrical disturbances [30,31].

## 4. RAS and ROS in Gitelman’s and Bartter’s Syndrome Patients

The overactivation of the RAS with concomitant reduced peripheral resistance, normal to low blood pressure and the absence of cardiovascular/renal remodeling, are the counterintuitive clinical features of GSBS patients (Figure 1B) [3,4].

Despite having elevated levels of Ang II, similar to those found in hypertensive subjects, GSBS patients have decreased α subunit of Gq protein (Gαq) expression, which as a result promotes intracellular Ca^2+^ release and PKC activation [3,32]. For example, in GSBS the decreased expression of Gαq influences the interplay between GDP dissociation and GTP binding; the subsequent PLCβ activation, which in turn elevates diacylglycerol (DAG) and IP3 production, leads to the increased release of intracellular Ca^2+^ [3,33]. The altered AT1R downstream signaling in GSBS is also modulated by the regulators of G-protein signaling (RGS) [34]. These proteins are critical for the G protein-coupled receptor (GPCR) activity because they control the signaling via GPCR by regulating a variety of effector proteins [3,35]. RGS can inhibit GPCR signaling in several ways: by accelerating GTP hydrolysis (GTPase activating protein-GAP) that turns off the GPCR signal; by decreasing GPCR sensitivity to its agonists; and by boosting signal decay after the GPCR agonist removal. Interestingly, and again in contrast to hypertensives who display decreased RGS-2 expression [36], GSBS patients have increased gene and protein expressions of RGS-2 [34]. GSBS patients’ increased expression of RGS-2 reduces the expression of the Gα subunit, resulting in reduced PLCβ activity, IP3 and DAG [3,34,37]. Therefore, Ca^2+^ release is reduced and less Ca^2+^-calmodulin (Ca^2+^/CaM) complex is formed. In addition, DAG reduces PKC activity, with consequent increased eNOS expression and NO production, resulting in reduced vascular tone [3] (Figure 1B). These intracellular pathways alter muscle contraction and peripheral resistance by reducing the phosphorylation of the regulatory chain of myosin II [3]. The activation of the G-protein RhoA and its effector Rho kinase (ROCK) regulates the phosphorylation state of the MYPT-1, which is the regulatory subunit of the myosin light chain phosphatase (MLCP) [35,38,39].

The RhoA/ROCK pathway in GSBS is downregulated, which reduces phosphorylated MLC and produces vasodilation [3,40,41]. In contrast, the RhoA/ROCK pathway is upregulated in hypertension, where it induces vasoconstriction [3,41]. In addition, in GSBS the expression of RhoA regulators is altered upstream as they have reduced expression of Rho guanine nucleotide exchanger factors (RhoGEFs) in association with increased guanine nucleotide dissociation inhibitor (RhoGDI) that inhibits the dissociation of GDP from GDI [3,40]. This is in contrast to the increased expression of p63RhoGEF and p115RhoGEF found in hypertensive patients [40,41]. In this way, when RhoA is in its inactive form, the upstream signaling for ROCK results is blunted.

A further feature of GSBS is the increased eNOS generated vasodilator NO, despite the increased activation of RAS, but consistent with reduced oxidative stress [3]. eNOS is known to be negatively regulated by PKC, a kinase activated via G protein signaling. However, as noted, GSBS patients’ G protein signaling is altered, which reduces activated PKC, thereby increasing eNOS activation with ensuing increased NO production [3] (Figure 1B). Moreover, the response of NOXs to Ang II is reduced in GSBS patients. The stimulation of monocytes isolated from GSBS patients with Ang II in vitro shows reduced p22^phox^ gene expression and TGFβ activity, which demonstrates reduced oxidative pathway activity in these patients [3,42].

Altogether, the repeated findings in GSBS patients that are the inverse of the pathway responses in hypertensives show that GSBS patients should be considered the mirror image of hypertension. Therefore, these patients provide a very useful human model to explore, identify and interrogate the roles played by RAS and oxidative stress in cardiovascular pathology and renal remodeling (Table 2).

## 5. Conclusions

The study of GSBS patients has identified multiple oxidant-related systems which suggest several potential therapeutic targets to reduce and/or block Ang II-related as well as other RAS- and ROS-related processes. As detailed above, the complex array of altered pathways and ROS-related effects found in GSBS clearly must combine to block the pathological effects typically associated with an elevated Ang II. The myriad of oxidants, as well as changes in their levels, alongside the multiple pathways, organelles and organ systems involved found in GSBS patients, provides a rationale for why treatments aimed solely at “oxidants” have proven mostly unsuccessful on clinical grounds [43,44]. However, blocking multiple Ang II dependent signals, such as via blocking AT1R and ROCK activity, for example, does lead to the improvement of cardiovascular–renal remodeling, in part via a reduction of oxidative stress [18,45,46]. In summary, GSBS as a human model obverse that of hypertension has provided and will continue to provide important clues as to new targets, as well as improved treatment regimens in hypertension and its long term complications, aimed at Ang II and oxidative stress mediated cardiovascular renal remodeling.

## Figures and Tables

**Table 1:** Description of mutations in GS and BS and their relative sequelae [1,2].

	GS	BS Type 1	BS Type 2	BS Type 3	BS Type 4	BS Type 4b	BS Type 5
Gene	SCL12A3	SCL12A1	KCNJ1	CLCNKb	BSND	CLCNKa+CLCNKb	MAGE-D2
Protein	NCC	NKCC2	ROMK	CIC-Kb	Barttin	CIC-Ka+CIC Kb	MAGE-D2
Region of the Tubule	DCT	TAL	TAL+DCT	TAL+DCT	TAL+DCT	TAL+DCT	TAL+DCT
Onset	Adulthood	Prenatally	Prenatally	0–5 years	Prenatally	Prenatally	prenatally
Features	HypoMg, HypoCa, HypoK, Mild dehydration	Polyuria HypoCl, HypoK, Alkalosis HyperCa nephrocalcinosis	Polyuria hypoCl, alkalosis, Transient HyperK, HyperCa	Failure to thrive HypoCl Alkalosis HypoK, HyperCa HypoMg	Polyuria hypoCl alkalosis HypoK, HyperCa deafness	Polyuria hypoCl alkalosis HypoK, HyperCa deafness	Polyuria, hypoCl, alkalosis, HypoK, HyperCa

HypoMg: hypomagnesemia; HypoK: hypokaliemia; HypoCa: hypocalciuria; HypoCl: hypochloremia; HyperCa: hypercalciuria; HyperK: hyperkaliemia.

**Table 2:** Comparison between Ang II signaling effects on GSBS and Hypertension.

Angiotensin II Effects	GSBS	Hypertension
PKC expression and activity	Decreased	Increased
Intracellular IP3 levels	Decreased	Increased
Intracellular Ca^2+^ release	Decreased	Increased
NO system	Increased	Decreased
eNOS expression	Increased	Decreased
ROCK activity (MYPT-1)	Decreased	Increased
RGS-2 expression	Increased	Decreased

## Data Availability

The data is contained within the article.
